# Characterizations of sulfate-reducing bacteria biofilm formed on N80 carbon steel in artificial shale gas field produced water

**DOI:** 10.1038/s41598-025-04332-6

**Published:** 2025-07-01

**Authors:** Lincai Peng, Shaomu Wen, Lei Yu, Hongjie Li, Feng Wang, Xi Deng

**Affiliations:** 1 Research Institute of Natural Gas Technology, PetroChina Southwest Oil & Gasfield Company, Chengdu, 610213 China; 2 PetroChina Southwest Oil and Gasfield Company, Chengdu, 610051 China

**Keywords:** Microbiologically influenced corrosion, SRB corrosion, Biofilm, Shale gas, Energy science and technology, Engineering, Materials science

## Abstract

The corrosion of steel caused by sulfate-reducing bacteria (SRB) has been a big trouble resulting in the service failure of engineering equipment, and SRB biofilm is the direct reason leading to the corrosion acceleration. In this work, SRB biofilms formed on N80 carbon steel in an artificial shale gas field produced water with different test conditions were characterized carefully by scanning electron microscopy (SEM), energy dispersive X-ray spectroscopy (EDS), fluorescence microscope, and three-dimensional stereoscopic microscope. Results demonstrate that test time, temperature, and initial SRB cell concentration can influence the growth and surface morphology of biofilm, and test time and temperature are primary factors. There is a highest corrosion rate of 0.100 ± 0.005 mm/y on the seventh day due to the high biological activity, and then corrosion rates gradually decline with time. The formed biofilms at different time have a similar morphology and the contents of elemental S in biofilms are high also suggesting SRB corrosion. Temperature can influence the biological activity of SRB, and then affect the formation of SRB biofilms. SRB has a higher biological activity at 20 and 37 °C than that of at 60 and 80 °C. The influence of initial SRB cell count differences on biofilm is weak.

## Introduction

Recent years, shale gas extraction from shale rock formations has a fast development in the world because shale gas is a clean energy, and United States and China have abundant shale gas resource which is helpful to cope with the growing energy demand^1–4^. However, the service failure of pipeline steel caused by corrosion is a big threat for the safety running of shale gas extraction^5–7^. Due to the complex and hostile environments in shale gas wells, the corrosion behavior and process of steel will much more complicated and changeable, and CO_2_ corrosion, H_2_S corrosion and under-deposit corrosion and so on are commonly found^8–10^. Furthermore, large amounts of bacteria have been found in shale gas produced water, especially sulfate-reducing bacteria (SRB), and the existence of bacteria can significantly participate and accelerate steel corrosion, called microbiologically influenced corrosion (MIC)^11,12^.

In recent years, MIC study has a fast development due to more and more concerns from corrosion researchers. Bacteria can survive well, and an abundance of bacteria (around 10^10^ cells/mL) in the Pinedale shale gas field have been found leading to the formation of black water as well as severe corrosion issues^13,14^. MIC has been a big trouble in shale gas fields have a deep influence on the integrity of pipelines and other facilities. Because localized corrosion is the primary type of MIC, localized corrosion, such as pitting corrosion, can easily cause pipeline leakage^15,16^. SRB has been found as the primary corrosive bacteria in shale gas environments accelerating steel corrosion^17^. SRB, a typical anaerobic bacteria, are found commonly exists in various environments, such as oil and gas produced water, seawater, and soil, and they can use sulfate as the terminal electron acceptor^18,19^. WU et al.^20^ analyzed the failure mechanism of shale gas gathering pipelines in the southern Sichuan Basin of China and found that SRB is one of the primary factors leading to the shale gas pipeline steel corrosion and the subsequent perforation.

Studies of SRB corrosion behavior and mechanism have been hot topics in recent years, and SRB can directly or indirectly enhance steel corrosion^10^. The direct way is the direct electron transfer from steel to SRB cells, while the indirect way is related to metabolic products, such as extracellular polymeric substances (EPS)^21–23^. However, the real SRB corrosion mechanism is still unclear due to its complexity. Especially in some rigorous environments, such as shale gas fields, SRB corrosion studies need to pay much more attention. In our previous research, it is found that temperature is a critical factor influencing corrosion in shale gas environment, with high temperatures typically promoting non-biological corrosion^24^. However, under high-temperature conditions, the biological activity and corrosive behavior of microorganisms undergo changes^25^. Elevated temperatures may cause a rapid decline in microbial activity, thereby mitigating MIC, but once microorganisms adapt to high-temperature environments, temperature and microbes may synergistically accelerate steel corrosion^11^. Therefore, the impact of temperature on MIC caused by SRB requires further in-depth research. Furthermore, SRB corrosion is closely related to the biofilm, and the formation of biofilm is the direct factor leading to the corrosion acceleration. Biofilm as the place resulting in corrosion, plays the primary role in corrosion^26–28^. However, the characterizations of SRB biofilm are still poor which is an important factor leading to the understanding of SRB corrosion.

In this work, N80 carbon steel was selected for this study because it is the most widely employed material for downhole tubing in shale gas fields. The corrosion of N80 steel caused by SRB was studied in a shale gas environment, and the formed SRB biofilms were deeply characterized by scanning electron microscopy (SEM), energy dispersive X-ray spectroscopy (EDS), fluorescence microscope, and three-dimensional stereoscopic microscope. This work firstly aims to investigating the effects of experimental time, initial cell concentrations, and temperature on the attachment and growth of SRB and subsequent biofilm formation, thereby revealing the corrosion behavior of SRB on steel and providing practical insights relevant to field applications in shale gas environments.

## Methods

### Materials

N80 carbon steel was used in this work, and it had a composition (wt%) of C 0.424, Mn 1.56, Si 0.384, P 0.009, S 0.007, Ni < 0.003, Cu 0.06, V 0.121, Mo < 0.004 and Fe balance. Steel specimens with a size of 40 mm × 10 mm × 2 mm were used to do biofilm characterizations and weight loss. All specimens were polished by grit silicon carbide papers of 400#, 800#, and 1200#. Subsequently, the specimens were washed with deionized water, acetone, and ethanol. Last, the specimens were radiated by an ultraviolet lamp for 30 min to do sterilization.

### SRB culturing and inoculation

Sulfate-reducing bacterium *Desulfovibrio caledoniensis* was used in this work, and SRB was cultured in a medium containing (g/L): K_2_HPO4 0.01, MgSO_4_·7H_2_O 0.2, NaCl 10, sodium lactate 4 mL, (NH_4_)_2_Fe(SO_4_)_2_ 0.2, and vitamin C 0.1. Before inoculation, SRB fresh culture medium was autoclaved at 121 °C for more than 20 min. Sparging N_2_ for 4 h to remove dissolved oxygen (DO). After inoculation, SRB was cultured at 37 °C to allow biofilm formation on the material surface. Testing methods of planktonic and sessile SRB cells was referred to the literature and their content was enumerated with the most probable number (MPN) method^29^.

### Test solution

An artificial shale gas produced water was prepared based on the components of field produced water of the Changning shale gas well of China, and it was composed (g/L) of KCl 5.2, NaCl 18, CaCl_2_ 0.8, Na_2_SO_4_ 0.3, MgCl_2_ 0.1, and NaCO_3_ 0.5. Furthermore, 10% SRB culture medium was added to the artificial shale gas produced water as the test solution. The pH value of test solution was 7.2.

### Weight loss measurements

The weight loss experiments were conducted by immering N80 carbon steel specimens in 250 mL of test solution at a constant temperature of 37 ± 1^o^C using a thermostatic water bath. After being immersed for different time, the specimens were taken out to do weight loss measurements. Initially, the surface corrosion products on specimens were removed by a pickling solution containing imidazoline derivative. Then the bare specimens were washed with deionized water, acetone, and anhydrous ethanol in series. Last, the specimens were dried using N_2_. All experiments were performed in triplicate to ensure the reproducibility of the experimental results. The error bars represent the standard deviation (± SD) calculated from three independent experimental replicates. The corrosion rates of specimens were calculated from the weight loss based on the following equation.

1 $$CR = \:\frac{87600 \times \Delta \text{m}}{\text{A} \times \rho \times \text{t}}$$

Where *CR*, *Δm*, and *A* correspond to corrosion rate (mm/y), specific mass changes (mg), and specimen area (cm^2^, respectively, and ρ and t assigned to the steel density (g/cm^3^) and test time (h).

### Characterizations of biofilm

ASEM (JSM-IT200, Jeol, Japan) and EDS were first used to observe the surface morphologies of biofilm and analyze the elemental composition of biofilm. Prior to SEM observation, the biofilm-covered specimens were taken out and immersed in a phosphate buffer solution containing 2.5 wt% glutaraldehyde to kill bacteria and fix the biofilm. Subsequently, the specimens were dehydrated in ethanol solution and dried using N_2_. Furthermore, a thin gold film was coated on the specimen surface to improve the electrical conductivity of the biofilm. A 3D stereoscopic microscope (Leica DVM6, Germany) was used to characterize the thickness of biofilm. A fluorescent dye (Molecular Probes™ FilmTracer™ LIVE/DEAD^®^ Biofilm Viability Kit) was used to stain the biofilm in darkness for 10 min, and then the stained biofilms were observed by a Leica fluorescence microscope (Leica Olympus FV-1000, Germany) to analyze the live and dead SRB cells in the biofilm. In the surface analysis test, the temperature of the unmarked part is 37 °C. All tests were repeated more than three times.

## Results

### Effect of test time on SRB biofilm

#### Weight loss

**Fig. 1:**
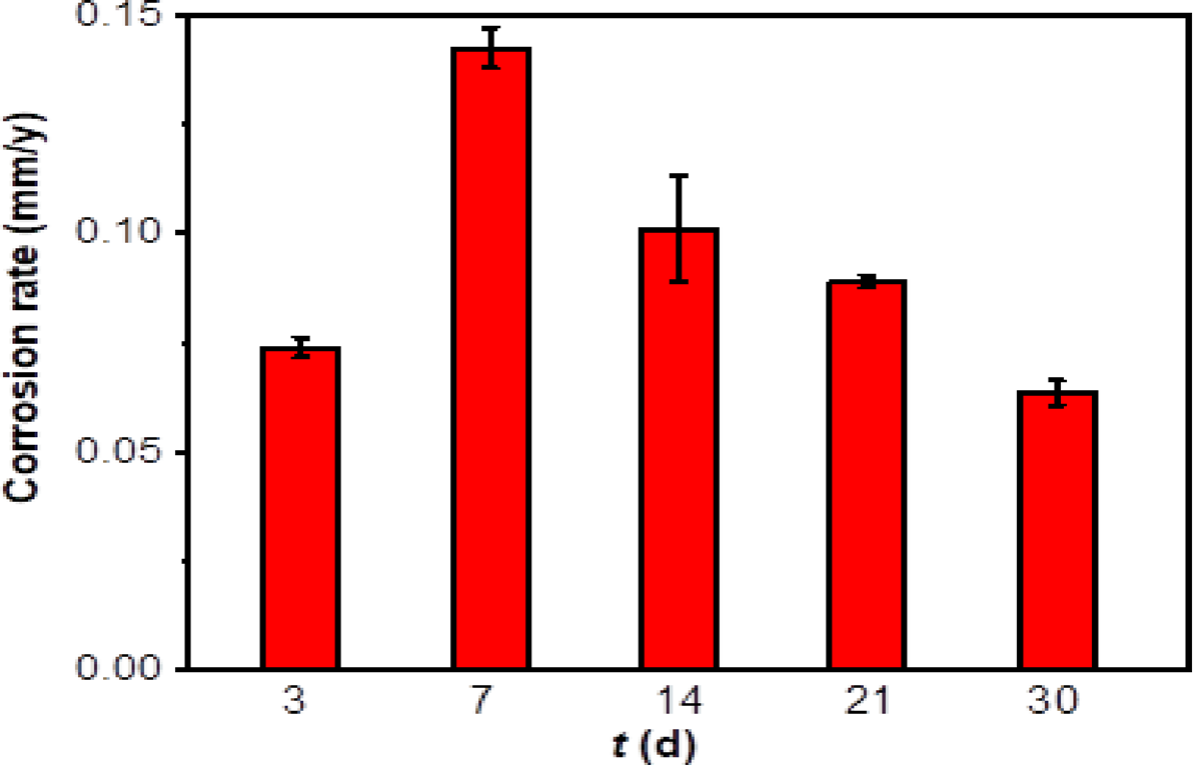
Corrosion rates of N80 carbon steel specimens calculated from weight loss immersed in artificial shale gas produced water containing SRB at different time.

The corrosion rates of specimens immersed in artificial shale gas produced water containing SRB at different time are shown in Fig. 1. It is seen that the corrosion rate of specimens in the early stage is small with a value of (0.070 ± 0.002) mm/y. The corrosion rate has the biggest value on 7th d, i.e., (0.100 ± 0.005) mm/y, and then the corrosion rates gradually decline with the increase of test time.

Therefore, it is concluded that SRB corrosion is related to the test time. In the early stage, the growth of SRB is in the adaptive phase, and their adsorption on the specimen results in slight corrosion due to incomplete biofilm and low biological activity^30^. On the 7th d, SRB has a high biological activity causing a high corrosivity against steel by accelerating the reduction of SO_4_^2−^ (Cationic reaction), thus then leading to the dissolution of Fe (Anodic reaction)^31^. After 14 d of testing, the biological activity of SRB will have a fast decreasing the consumption of nutrients, thus then leading to a decrease in steel corrosion rates.

2 $${\text{Anodic reaction}}:{\text{ Fe}} \to {\text{Fe}}^{{{\text{2}} + }} + {\text{2e}}^{ - }$$

3 $${\text{Cathodic reaction}}:{\text{SO}}_{{\text{4}}} ^{{{\text{2}} - }} + {\text{9H}}^{ + } + {\text{8e}}^{ - } \to {\text{HS}}^{ - } + {\text{4H}}_{{\text{2}}} {\text{O}}$$

#### SEM characterizations of biofilm

**Fig. 2:**
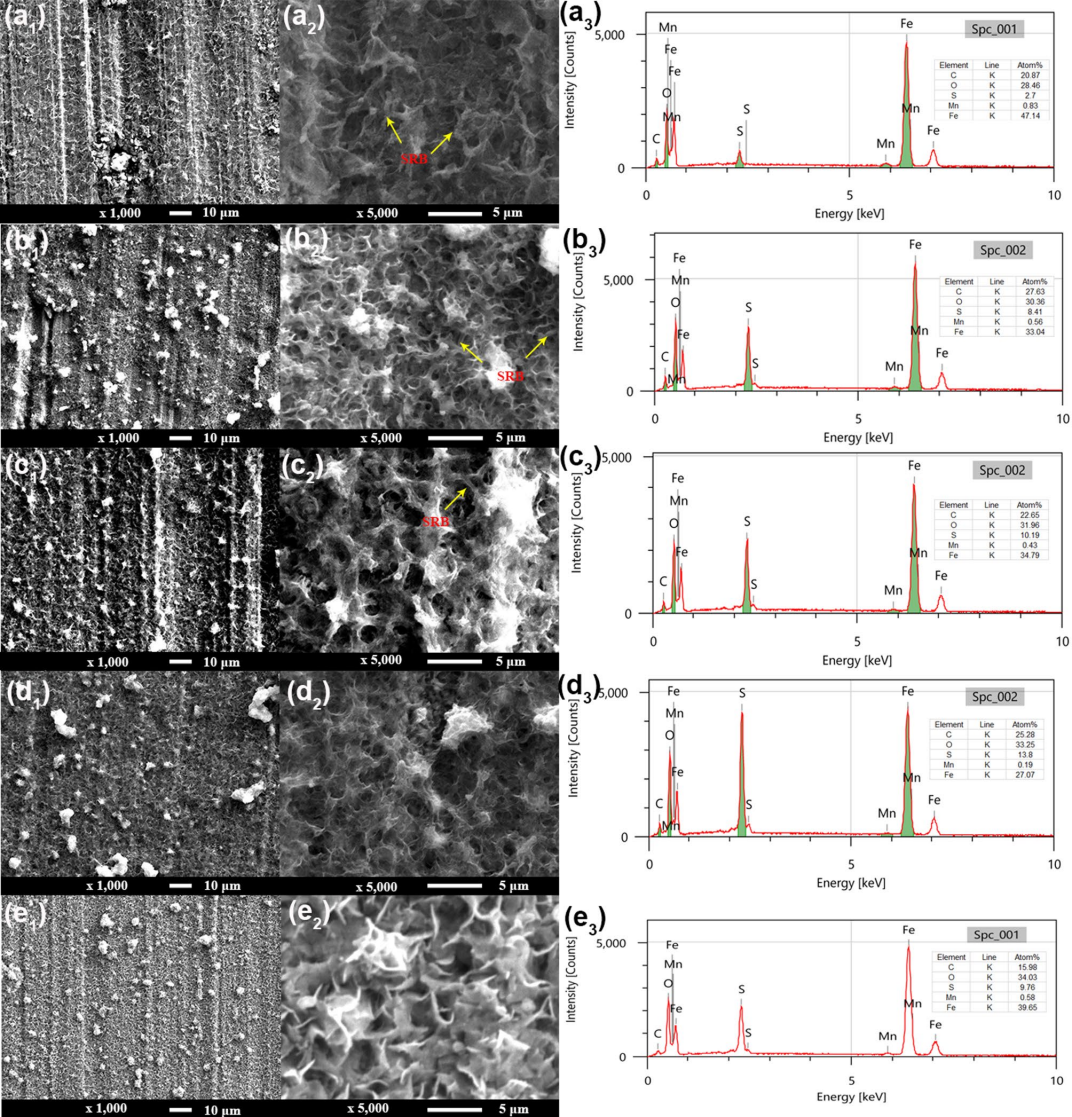
SEM images of biofilms and the corresponding EDS analysis after being immersed in artificial shale gas produced water containing SRB with different time: (a_1_, a_2_ and a_3_) 3 d, (b_1_, b_2_ and b_3_) 7 d, (c_1_, c_2_ and c_3_) 14 d, (d_1_, d_2_ and d_3_) 21 d, (e_1_, e_2_ and e_3_) 30 d.

Figure 2 shows the SEM images of biofilms and the corresponding EDS analysis after being immersed in artificial shale gas produced water containing SRB at different time. It is seen that the formed biofilms at different time have a similar morphology. Some SRB cells can be seen covered on the biofilms formed on 3rd and 7th d (Fig. 2a_2_ and b_2_), and EDS results also show a high content of elemental S (Fig. 2a_3_ and b_3_). The S content of biofilm on the 3rd is 2.7 at%, and it increases to 8.41at. % on the 7th d, suggesting that more Fe_x_S_y_ corrosion products are generated by SRB. The formation of Fe_x_S_y_ indirectly confirms SRB corrosion. With the increase of test time, the porous biofilms can be observed (Fig. 2b_2_, c_2_ d_2_, and e_2_), and meanwhile, the contents of elemental S also have an increase. On 30th d, some lamellar corrosion products can be observed (Fig. 2e_2_). Therefore, the surface morphologies of biofilms gradually change with the increase of test time and large amounts of Fe_x_S_y_ corrosion products are formed. Fe_x_S_y_ is the typical corrosion product of SRB^33^.

#### Biological activity of SRB biofilms

**Fig. 3:**
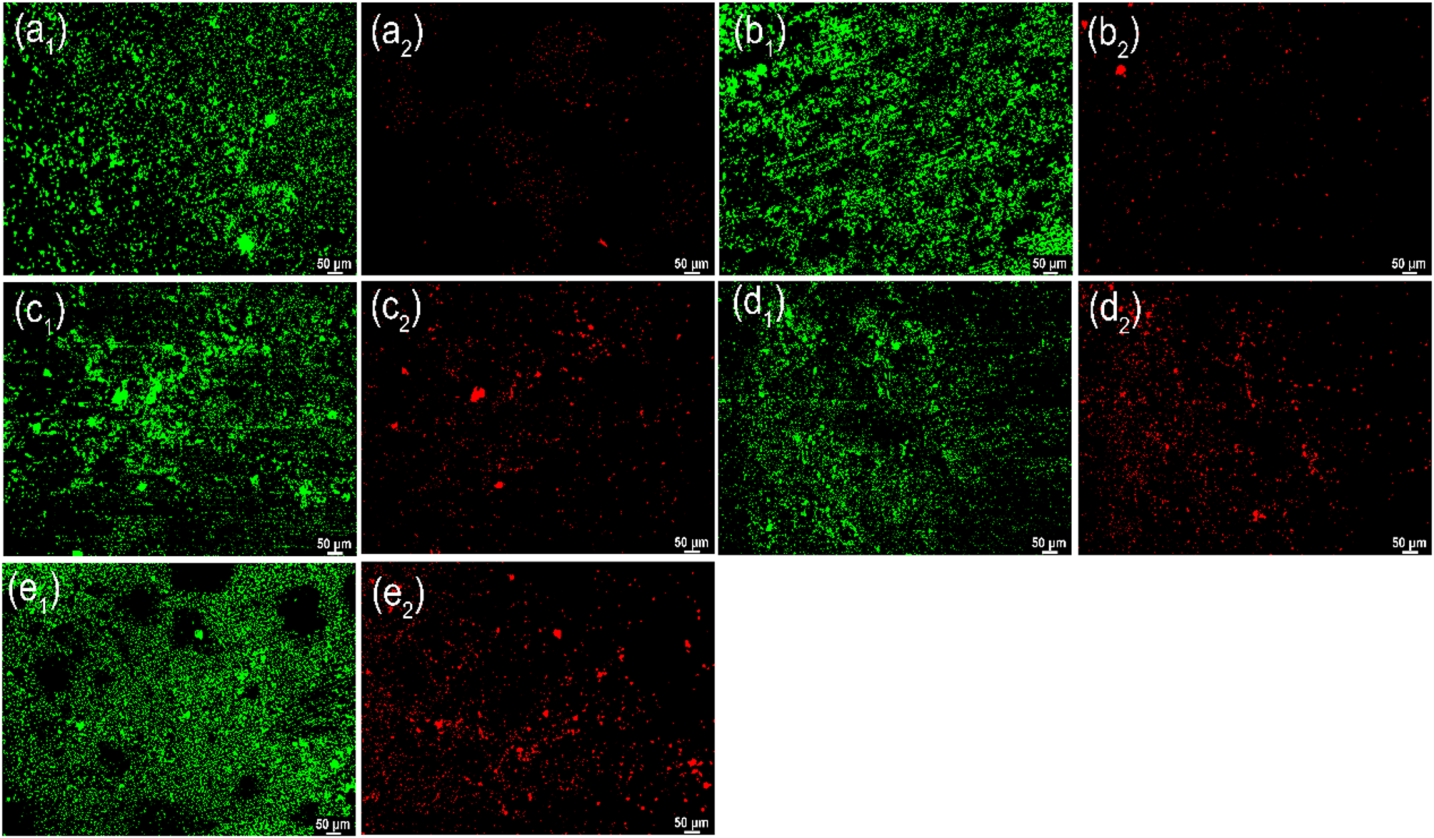
Live/dead staining of adherent SRB cells on the specimen surface after 3 (a_1_ and a_2_), 7 (b_1_ and b_2_), 14 (c1 and c2), 21 (d_1_ and d_2_), and 30 (e_1_ and e_2_) d of testing in artificial shale gas produced water containing SRB.

The live and dead SRB cells in biofilms have been characterized by a fluorescence microscope, and the images are presented in Fig. 3. Green and red colors of images in Fig. 3 indicate live and dead cells, respectively. The strong fluorescence intensity indicates the high SRB cell density and counts. On 3rd d, amounts of SRB have attached to the surface of the steel surface and only little dead SRB cells (Fig. 3a_1_ and a_2_). The live SRB cell density has an increase from 3rd to 7th d, and the dead SRB cells have a low density (Fig. 3b_1_ and b_2_). From 14th to 30th d, it is seen that the live SRB cell counts have a gradual decrease but the dead SRB cell counts increase gradually with time (Fig. 3c_1_–e_2_). It is also found that the distribution of SRB cells in biofilm is not heterogeneous, and they tend to agglomerated growth. The agglomeration of SRB cells is conducive to the growth and biological activity of SRB as well as the stability of SRB biofilm^33,34^. The cell counts (Table 1) in biofilm also show that the sessile SRB concentration has the biggest value on 7th d, and then gradually decreases with time, which is consistent with the analysis results in Fig. 3.

**Table 1:** The sessile SRB cell counts in biofilms after 3, 7, 14, 21, and 30 d of testing in artificial shale gas produced water containing SRB.

t (d)	3	7	14	21	30
Cell counts (cells/cm^2^)	1 × 10^5^	1 × 10^6^	1 × 10^4^	1 × 10^2^	1 × 10^1^

#### The change in biofilm thickness

**Fig. 4:**
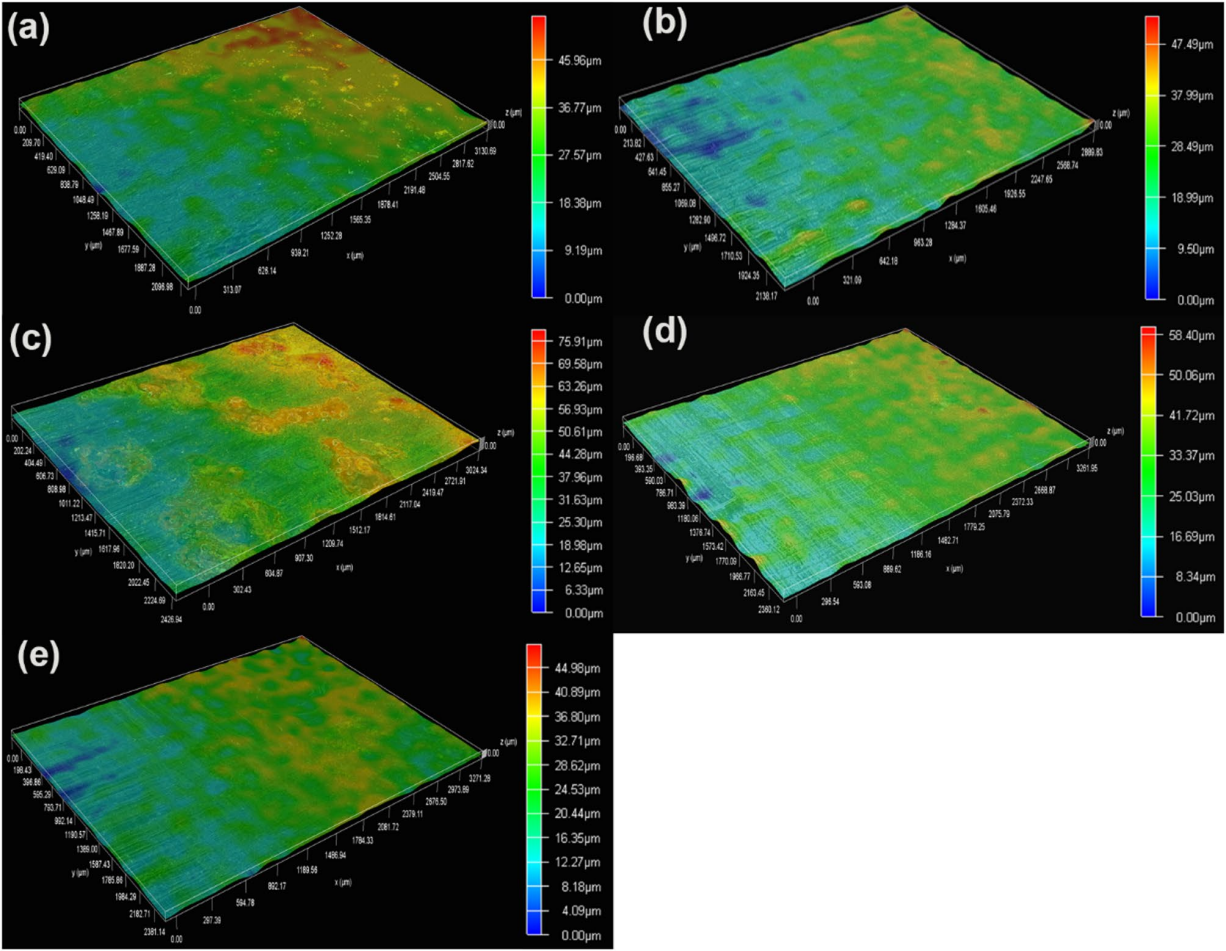
3D surface morphologies of biofilms formed on specimens in artificial shale gas produced water containing SRB with different time: (**a**) 3 d, (**b**) 7 d, (**c**) 14 d, (**d**) 21 d, (**e**) 30 d.

3D surface morphologies of biofilms formed on specimens in artificial shale gas produced water containing SRB at different time are presented in Fig. 4. Part of the biofilm is removed to measure the thickness of biofilm. It is seen that the formed SRB biofilms are heterogeneous, and the thickness of biofilm changes little from 3rd to 7th d (Fig. 4a and b). The thickness of biofilm reaches a biggest value on 14d, i.e., 75.91 μm. Subsequently, the thicknesses of SRB biofilm have decreased with time, and the thickness of biofilm is 44.98 μm on 30th d. The decrease of SRB biofilm during the later period is due to the part exfoliation of biofilm. Biofilm exfoliation commonly occurs due to the decrease of biofilm stability after a long time of incubation^35^. Sun et al. also found that SRB biofilm starts to exfoliate after 15 d of testing due to the decrease in SRB biological activity^36^. EDS analysis results also show the decrease of elemental S content in biofilm on 30th d which is probably due to the exfoliation of SRB biofilm. Therefore, test time is one of the primary factors influencing the formation of SRB biofilm and the subsequent SRB corrosion behavior. During the culture later period, the consumption of large amounts of nutrients directly causes a considerable decrease in biological activity and corrosivity of SRB^37,38^.

### Effect of temperature on SRB biofilm

#### SEM analysis of SRB biofilms formed at different temperature

**Fig. 5:**
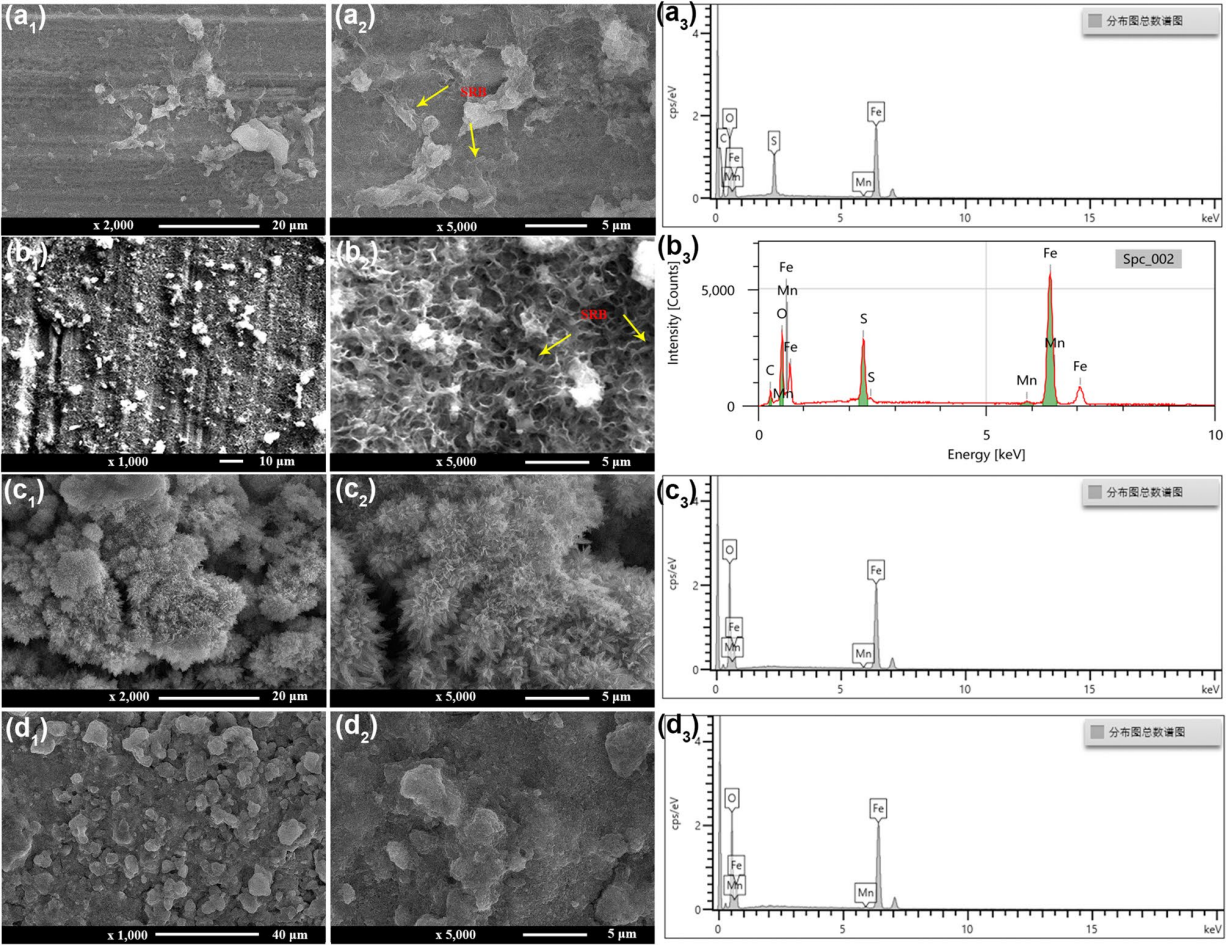
SEM images of SRB biofilms and the corresponding EDS analysis after 7 d of testing in artificial shale gas produced water containing SRB at different temperatures: (a_1_, a_2_ and a_3_) 20 °C, (b_1_, b_2_ and b_3_) 37 °C, (c_1_, c_2_ and c_3_) 60 °C, (d_1_, d_2_ and d_3_) 80 °C.

SEM images of SRB biofilms formed at different temperatures in artificial shale gas water after 7 d of testing and the corresponding EDS analysis are presented in Fig. 5; Table 2. At 20 °C, the formed SRB biofilm is compact, which is different from the SRB biofilm formed at 37 °C (Fig. 5a_1_ and b_1_). EDS results show a high content of elemental S in biofilms (Table 2), suggesting a good biological activity of SRB at 20 and 37 °C. However, the surface morphologies of SRB biofilms formed at 60 and 80 °C have a considerably big difference, and large amounts of corrosion product particles can be observed (Fig. 5c_1_ and d_1_). Furthermore, the elemental S is not detected in the biofilms formed at 60 and 80 °C, suggesting a low SRB biological activity. From EDS results, the corrosion products are mainly composed of iron oxides which can be due to the oxidation of corrosion products during the preparation of SEM samples. SEM images show that SRB corrosion behaviors have a big change with the increase in temperature. In abiotic conditions, high temperature can accelerate the reaction kinetics, and then leading to the corrosion acceleration of steel^39^. However, high temperature will cause a fast decrease in bacterial biological activity, which is negative to MIC acceleration^11,40^. Therefore, the abiotic corrosion can be the primary at 60 and 80 °C, but SRB corrosion is dominant at 20 and 37 °C.

**Table 2:** EDS analysis results of SRB biofilms formed at different temperatures corresponding to SEM images in Fig. 5.

Elements	C	O	Mn	Fe	S
20 °C (wt%)	11.98	22.35	0.62	56.97	8.08
37 °C (wt%)	27.63	30.36	0.56	33.04	8.41
60 °C (wt%)	–	27.34	0.6	72.06	–
80 °C (wt%)	–	25.13	1.08	73.79	–

“–“ indicates “not detected”.

#### Biological activity of SRB biofilms formed at different temperature

**Fig. 6:**
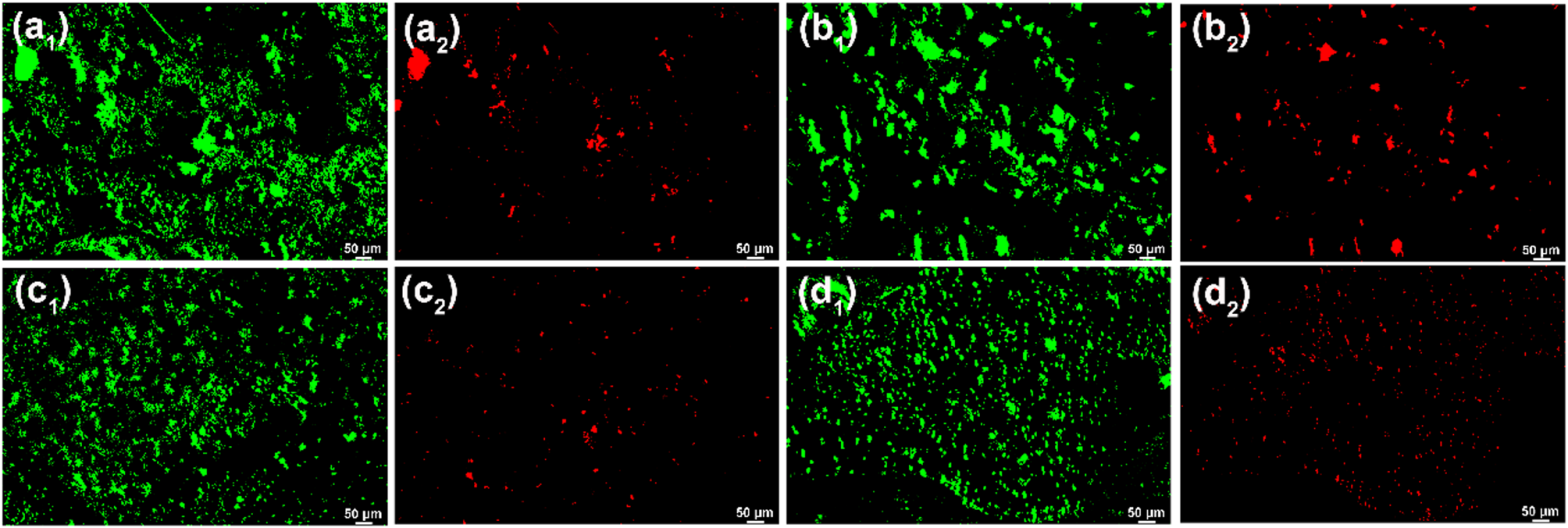
Live/dead staining of adherent SRB cells on the specimen surface after 7 d of testing in artificial shale gas produced water containing SRB at a different temperature: (a_1_ and a_2_) 20 °C, (b_1_ and b_2_) 37 °C, (c_1_ and c_2_) 60 °C, (d_1_ and d_2_) 80 °C.

Live/dead staining of adherent SRB cells on the specimen surface after 7 d of testing in artificial shale gas produced water containing SRB at different temperatures are shown in Fig. 6. It is seen that the biofilms formed at 20 and 37 °C contain large amounts of live and dead SRB cells (Fig. 6a_1_,b_2_), demonstrating that SRB can maintain a good biological activity at 20 and 37 °C. Therefore, SRB corrosion at 20 and 37 °C should be much more severe. However, the live and dead SRB cells decline apparently after the temperature increases to 60 and 80 °C (Fig. 6c_1_,d_2_), especially the dead SRB cells. SRB can survive at 60 and 80 °C but their biological activity has declined considerably. From Table 3, it is also observed that the sessile SRB cells in biofilms formed at 60 and 80 °C have low values, and the cell counts decline more than three orders of magnitudes compared to the sessile SRB cells at 37 °C. In conclusion, temperature is one of the important factors influencing the biological activity of SRB and the subsequent SRB corrosion. The high temperature is harmful to the metabolism and growth of SRB. However, once SRB has adapted to the high-temperature environment after a long time, the metabolism and growth of SRB will accelerate leading to the enhancement of SRB corrosion. MIC at a high temperature is also one of the important factors leading to the corrosion failure of equipment, such as pipelines^11,17,41^.

**Table 3:** The sessile SRB cell counts in biofilms after 7 d of testing in artificial shale gas produced water containing SRB at different temperatures.

Temperature (°C)	20	37	60	80
Cell counts (cells/cm^2^)	1 × 10^5^	1 × 10^6^	1 × 10^3^	1 × 10^2^

#### The thicknesses of SRB biofilms formed at different temperature

**Fig. 7:**
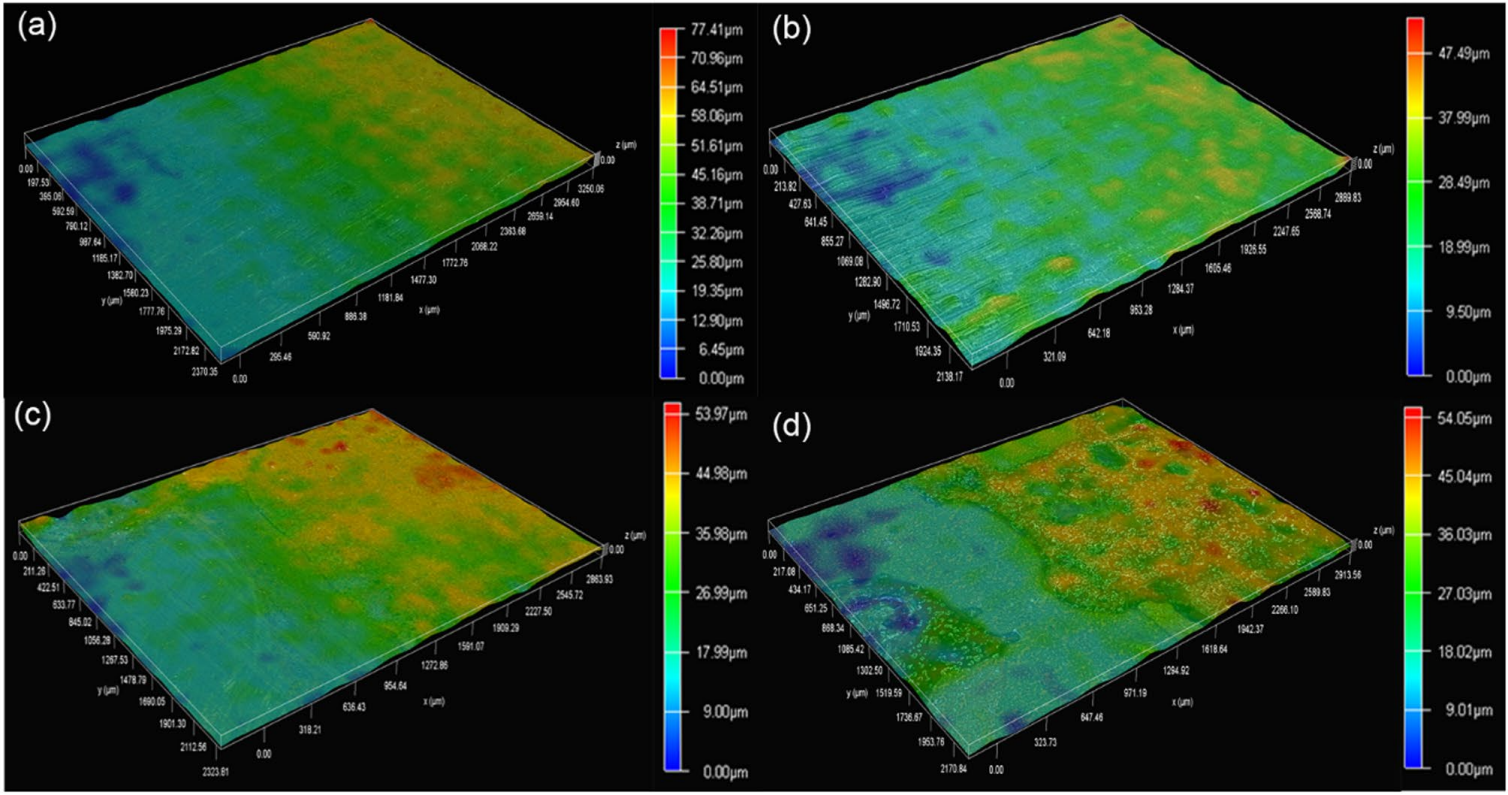
3D surface morphologies of biofilms formed on specimens after 7 d of testing in artificial shale gas produced water containing SRB at a different temperature: (**a**) 20 °C, (**b**) 37 °C, (**c**) 60 °C, (**d**) 80 °C.

3D surface morphologies of biofilms formed on specimens after 7 d of testing in artificial shale gas produced water containing SRB at different temperatures are depicted in Fig. 7. The surface morphologies of SRB biofilm formed at 20, 37, 60, and 80 °C have a big difference, and the more corrosion products can be observed on the specimens at 60, and 80 °C. The high temperature can promote abiotic corrosion, thus then leading to the formation of more corrosion products. Furthermore, all the biofilms look heterogeneous and their thicknesses at 20, 37, 60, and 80 °C are 77.41, 47.49, 5.97, and 54.05 μm, respectively. However, SRB biofilms are easily agglomerated at 20 and 37 °C leading to the formation of some small peaks, just like mountain peaks. These lead to small differences in the thicknesses of SRB biofilms formed at different temperatures. However, the effect of temperature on SRB corrosion is noticeable.

#### Effect of initial cell concentrations on SRB biofilm

**Fig. 8:**
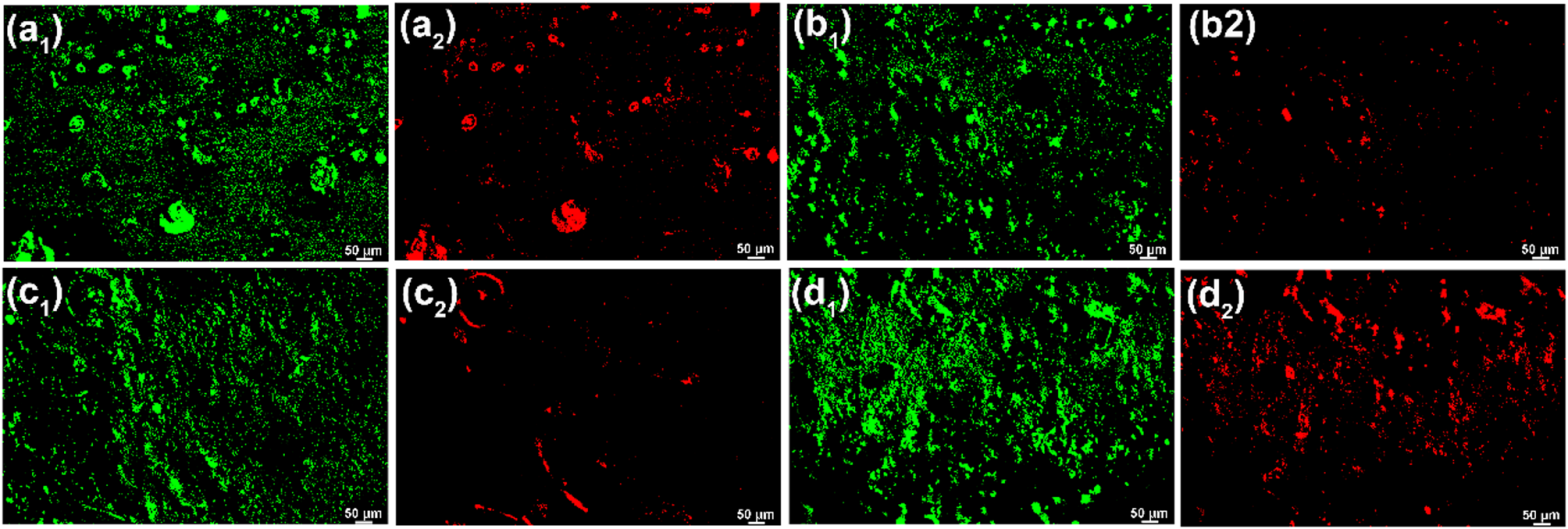
Live/dead staining of adherent SRB cells on the specimen surface after 7 d of testing in artificial shale gas produced water containing SRB at 37 °C with different initial cell concentrations: (a_1_ and a_2_) 10^1^ cells/mL, (b_1_ and b_2_) 10^2^ cells/mL, (c_1_ and c_2_) 10^3^ cells/mL, (d_1_ and d_2_) 10^4^ cells/mL.

**Fig. 9:**
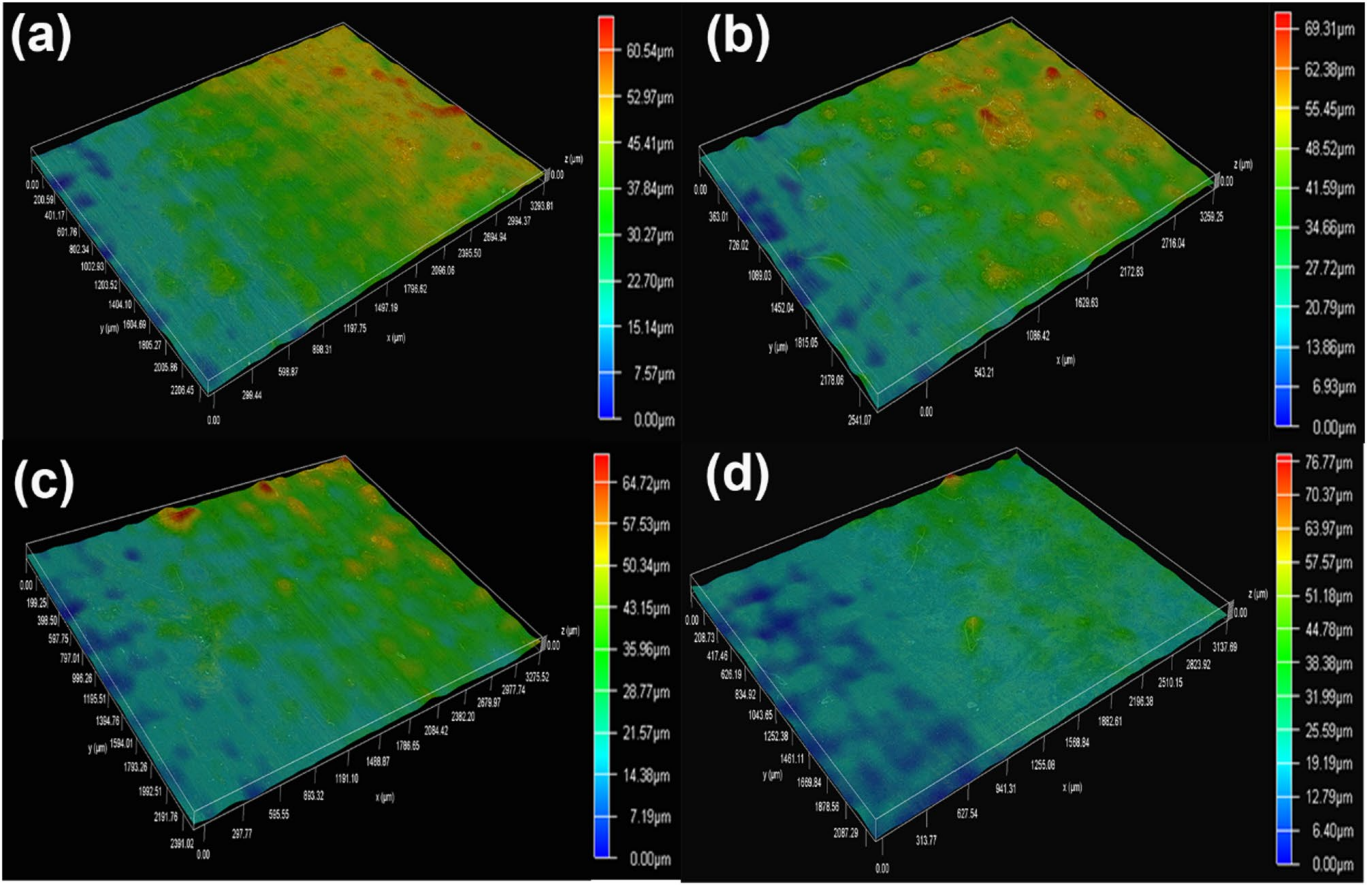
3D surface morphologies of biofilms formed on specimens after 7 d of testing in artificial shale gas produced water containing SRB at 37 °C with different initial cell concentrations: (**a**) 10^1^ cells/mL, (**b**) 10^2^ cells/mL, (**c**) 10^3^ cells/mL, (**d**) 10^4^ cells/mL.

Live/dead staining of adherent SRB cells on the specimen surface after 7 d of testing in artificial shale gas produced water containing SRB at 37 °C with different initial cell concentrations are shown in Fig. 8. There are large amounts of live and dead SRB cells in biofilms formed with different initial SRB cell concentrations. Therefore, the initial cell concentrations have little effect on the biofilm formation because SRB has a high adaptive ability and they can have fast growth during the logarithmic phase. Once SRB enters the stable phase, the influence of initial cell count differences on biofilm formation can be small. The corresponding 3D surface morphologies of biofilms formed with different initial SRB cell concentrations can be seen in Fig. 9. The surface morphologies of SRB biofilms look similar, and their thicknesses are also close, suggesting that the effect of initial SRB cell concentrations on biofilm formation is poor.

It should be pointed out that the factors influencing the adhesion and growth of SRB and the subsequent formation are not only test time, temperature, and initial cell concentration. The other factors, such as salinity, degree of mineralization, and water quality, can potentially influence SRB biological activity and the subsequent biofilm formation. However, the related studies are poor. Salinity, degree of mineralization, and water quality usually can keep a stable state for the specific shale gas wells. Therefore, in this work, test time, temperature, and initial cell concentration as the primary factors are studied to investigate their effect on SRB biofilm. From the above results, it is seen that test time and temperature have a deep influence on SRB biofilm, especially since temperature can change SRB biological activity. Once the biological activity of SRB has an apparent decrease, the corrosion type will change from MIC to abiotic corrosion, which means that the corrosion behaviors and mechanism of steel will change at the same time. For MIC studies, the good biological activity is the basis for the biofilm formation^42,43^. Biofilms are usually composed of bacterial cells, extracellular polymeric substances (EPS), corrosion products nucleic acid, etc^44,45^. The specific extraction of biofilm components and the subsequent analysis is also very important, but the extraction, such as EPS, is also very difficult due to its low content in biofilm. However, this will be carefully studied and overcome in our future works.

## Conclusions

SRB corrosion of N80 carbon steel in a shale gas environment is serious, and test time is one of the important factors influencing steel corrosion caused by SRB. The corrosion rate has the biggest value of (0.100 ± 0.005) mm/y on 7th d due to the high biological activity, and then the corrosion rates gradually decline with the increase of test time. The surface morphologies of SEM images show that the formed biofilms at different time have similar morphology, and high contents of elemental S can be found suggesting the formation of Fe_x_S_y_, the typical corrosion products of SRB. The content of elemental S in the biofilm formed at 21 d is the highest with a value of 13.8at.%. The high elemental S contents in SRB biofilms suggest that SRB can attach to steel well and cause the formation of biofilm. SRB biofilms have a higher biological activity on 7th d, and the content of dead SRB cells increases with time. The thicknesses of SRB biofilm reach the biggest value on 14 d, i.e., 75.91 μm. The decrease in SRB biofilm thicknesses demonstrates the existence of part exfoliation biofilm after a long incubation time probably due to the decrease in SRB biological activity.

Temperature has a deep influence on the biological activity and the growth of SRB biofilm. SRB can grow well at 20 and 37 °C, and keep a good biological activity. However, the biological activity of SRB has a fast decrease at 60 and 80 °C but small amounts of SRB still can survive, thus then causing a big difference in surface morphologies. The abiotic corrosion can be primary at 60 and 80 °C, but SRB corrosion is dominant at 20 and 37 °C. SRB biofilms are easily agglomerated at 20 and 37 °C while the biofilms formed at 60 and 80 °C are much more flat. Furthermore, the effect of initial SRB cell concentrations on biofilm is also investigated, but the influence of initial SRB cell concentration differences is small. SRB can have a fast growth and reach a high concentration even if the initial SRB cell count is low.

## Data Availability

The corresponding author has all the data connected to this work. All data or inquiries connected to this study can be acquired by contacting the corresponding author.
